# Spatio-temporal modeling of signaling protein recruitment to EGFR

**DOI:** 10.1186/1752-0509-4-57

**Published:** 2010-05-06

**Authors:** Ming-yu Hsieh, Shujie Yang, Mary Ann Raymond-Stinz, Jeremy S Edwards, Bridget S Wilson

**Affiliations:** 1 Department of Electrical and Computer Engineering, University of New Mexico, 1 University of New Mexico, Albuquerque, NM 87131, USA; 2 Department of Pathology, University of New Mexico Health Sciences Center, 1 University of New Mexico, Albuquerque, NM 87131, USA; 3 Department of Molecular Genetics and Microbiology, University of New Mexico Health Sciences Center, 1 University of New Mexico, Albuquerque, NM 87131, USA and Chemical and Nuclear Engineering, University of New Mexico, 1 University of New Mexico, Albuquerque, NM 87131, USA; 4 The University of New Mexico Cancer Research and Treatment Center, University of New Mexico Health Sciences Center, 1 University of New Mexico, Albuquerque, NM, 87131, USA

## Abstract

**Background:**

A stochastic simulator was implemented to study EGFR signal initiation in 3D with single molecule detail. The model considers previously unexplored contributions to receptor-adaptor coupling, such as receptor clustering and diffusive properties of both receptors and binding partners. The agent-based and rule-based approach permits consideration of combinatorial complexity, a problem associated with multiple phosphorylation sites and the potential for simultaneous binding of adaptors.

**Results:**

The model was used to simulate recruitment of four different signaling molecules (Grb2, PLCγ1, Stat5, Shc) to the phosphorylated EGFR tail, with rules based on coarse-grained prediction of spatial constraints. Parameters were derived in part from quantitative immunoblotting, immunoprecipitation and electron microscopy data. Results demonstrate that receptor clustering increases the efficiency of individual adaptor retainment on activated EGFR, an effect that is overridden if crowding is imposed by receptor overexpression. Simultaneous docking of multiple proteins is highly dependent on receptor-adaptor stability and independent of clustering.

**Conclusions:**

Overall, we propose that receptor density, reaction kinetics and membrane spatial organization all contribute to signaling efficiency and influence the carcinogenesis process.

## Background

The ErbB or Epidermal Growth Factor Receptor (EGFR) family of receptor tyrosine kinases consists of four members: EGFR (ErbB1), ErbB2, ErbB3, and ErbB4. Under normal physiological conditions, they propagate signals regulating cell proliferation, differentiation, motility and apoptosis. Changes in expression and aberrant activation, especially of EGFR and ErbB2, are associated with a variety of cancers [1]. Upon ligand binding, EGFR undergoes a conformational change that leads to the formation of homodimers (EGFR-EGFR) and heterodimers (i.e., EGFR-ErbB2) [2]. Dimerization induces kinase activation and transphosphorylation of multiple tyrosine residues in receptor cytoplasmic tails [3-5]. The phosphotyrosine residues serve as docking sites for a large number of cytoplasmic adaptor proteins and enzymes [6]. For a given cell type, the specificity and potency of EGFR-mediated intracellular signaling is mediated by the cell's repertoire of phosphotyrosine-binding proteins recruited to the EGFR cytoplasmic tail.

In this work, we use an agent-based model to evaluate the effects of reaction kinetics, steric constraints and receptor clustering on the docking of four EGFR binding partners: Grb2, Shc, Stat5 and PLCγ1. The adaptor Grb2 lacks enzymatic activity and consists of one Src homology (SH) 2 domain and two SH3 domains [7]. Its SH2 domain docks to specific EGFR phosphotyrosine residues and its SH3 domains bind to a Ras guanine nucleotide exchange factor, Sos [8,9]. The adaptor Shc also binds directly to activated EGFR by two distinct phosphotyrosine interaction domains, an NH2-terminal phosphotyrosine binding (PTB) domain and a COOH-terminal SH2 domain [10,11]. Recruitment of Grb2 and Shc lead to activation of ERK (extracellular signal regulated kinase) [12], which translocates into the nucleus and induces gene expression [13]. The transcription factor Stat5 is activated by phosphorylation after docking to EGFR or indirectly through Src-mediated EGFR signaling [14,15]. Activated Stat5 translocates into the nucleus where it regulates the transcription of selected genes involved in oncogenesis [16,17]. PLCγ1 has two SH2 domains, one SH3 domain and two pleckstrin homology (PH) domains [18]. It is recruited to phosphorylated EGFR through its SH2 domains, where it serves as a substrate for EGFR kinase activity. Tyrosine phosphorylation of PLCγ1 then leads to an increase in its enzyme activity [19]. PLCγ1 pathway plays a significant role in EGFR-mediated cell signaling, including calcium signaling [20], receptor endocytosis [21] and cell motility [22]. Overexpression and hyperactivation of PLCγ1 has been implicated in breast and prostate cancers, and has especially been linked to cancer cell invasion [23,24].

The process of signaling through ErbB receptors involves highly connected networks of interacting components. Improved understanding of receptor signaling through systems biology approaches has a number of potential practical applications, such as the rational design of drugs to treat cancer [25]. The accuracy of mathematical models relies heavily on quantitative characterization of signaling components and their interactions, such as measurement of expression levels and reaction rate constants. However, the acquisition of quantitative information is no small task, in part because signaling proteins contain multiple phosphorylation sites and may interact with multiple binding partners. Many groups have studied the affinity between EGFR phosphopeptides and the binding domains of Grb2, Shc, STATs, and PLCγ1 using protein microarrays [26], Surface Plasmon Resonance (SPR) [27-29] and Isothermal Titration Calorimetry (ITC) [30,31]. These studies provided estimates of dissociation equilibrium constants (K_d_) but association and dissociation rate constants of the reactions were typically either not measured or derived indirectly. Moreover, none of these measurements were based upon whole EGFR within lipid bilayers. To understand distinct recruitment behaviors for the different signaling proteins, it is important to arrive at better estimates of their association and dissociation kinetics. This will require new experimental and computational approaches. In an recent experimental development, Morimatsu and colleagues applied single molecule analysis to measure the reaction rate constants of Grb2 with membranes bearing intact, phosphorylated EGFR [32]. In this study, we combined several quantitative experimental approaches, including western blotting analysis and semi-quantitative electron microscopy, to evaluate the kinetics of EGFR phosphorylation and adaptor recruitment to the plasma membrane of EGF-stimulated cells. Rate constants for EGFR phosphorylation/dephosphorylation and adaptor docking are estimated by fitting this data to simulations in our agent-based stochastic model, Signaling Pathways Simulator (SPS) [33].

Our model specifically considers the phenomenon referred to as combinational complexity, which has been a challenging problem for deterministic mathematical models that employ differential equations to describe cell signaling pathways [34,35]. For example, because the EGFR becomes phosphorylated on at least nine tyrosine residues during signaling, there are more than 260,000 distinct combinations of these phosphoforms for a dimer of EGFR. Additional molecular diversity can arise when accounting for potential simultaneous interactions of receptor tails with multiple cytoplasmic adaptors. Previous models of ErbB signaling reduce the problem of combinatorial complexity by making several assumptions, including simultaneous phosphorylation and dephosphorylation of receptor tyrosine residues, representation of all tyrosine residues as a single 'virtual phosphorylation site', and exclusion of multiple cytoplasmic adaptors on the same receptor tail based upon competitive binding [34,36-39]. In an important advance, Blinov and colleagues developed a rules-based model of early EGFR signaling events, capable of evaluating more than 300 molecular species connected through ~4000 unidirectional reactions [35]. Our spatial stochastic model is also "rules-based" and specifically designed to consider largely unexplored contributions of 1) EGFR clustering [33,40,41] and anomalous diffusion [42], 2) distinct temporal patterns of EGFR tyrosine phosphorylation and 3) the potential for multiple adaptors to bind to the same phosphorylated EGFR tail. We refer to the latter concept as "sharing" and base our simulation rules upon the results of coarse-grain molecular docking modeling. In SPS, receptors diffuse in the two dimensional plasma membrane. Rules established for diffusion in and out of defined subdomains of the membrane (protein islands or rafts) provide a mechanism for receptor clustering [33]. We demonstrate that the agent-based spatial model can effectively address problems associated with combinational complexity and make testable predictions about adaptor binding and signaling output that are consistent with novel, quantitative experimental data sets. The simulation results suggest that adaptor sharing is highly dependent on reaction kinetics. The spatial model also predicts receptor clustering results in more efficient adaptor retainment, particularly at normal receptor expression levels.

## Results

### **Coarse-grained molecular docking simulations establish rules for competitive or simultaneous adaptor recruitment to the EGFR cytoplasmic tail**

For convenience's sake, conventional models typically assume that the docking of adaptor proteins is a competitive process [34,36,37,43]. Nevertheless, it is possible that neighboring phosphotyrosine residues on the EGFR tail can recruit distinct proteins at the same time [26], a phenomenon we refer to hereafter as "sharing". In theory, the ability of multiple proteins to dock on the same tail could influence signal transduction efficiency. To address this in our stochastic model, we first sought to establish docking "rules" based upon coarse-grained molecular docking methods.

**Figure 1:**
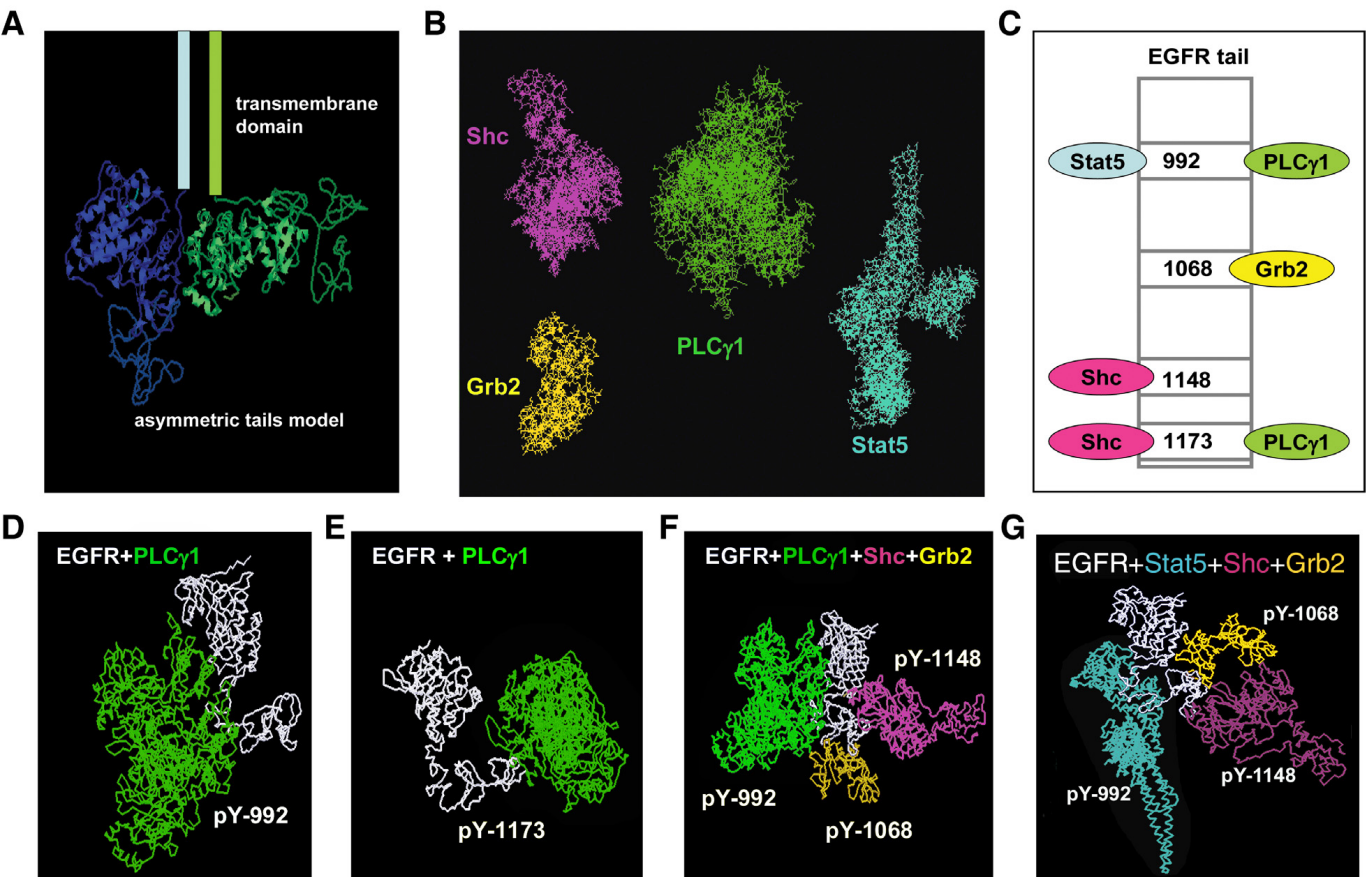
**Hierarchy of EGFR tail binding partners predicted by coarse-grained molecular modeling method**. (**A**) Coarse-grained molecular modeling of EGFR C-terminal tail attached to the asymmetric model of EGFR kinase domain obtained from Zhang et al [45]. (**B**) Full-length coarse-grained molecular models of Shc (purple), Grb2 (yellow), PLCγ1 (green), and Stat5 (cyan). (**C**) Simplified systematic interaction profiling of the EGFR tyrosine family used in our study. In this model, EGFR has four cytoplasmic interaction partners, one binding site for each of Stat5 and Grb2, and two binding sites for each of Shc and PLCγ1. Coarse-grained docking method was used to develop the hierarchy of EGFR tail binding partners. Some possible docking methods between the four adaptors and EGFR include (**D**) PLCγ1 docked to pY992, (**E**) PLCγ1 docked to pY1173, (**F**) PLCγ1, Shc, and Grb2 simultaneously docked to EGFR at pY992, pY1148, and pY1068, respectively, and (**G**) Stat5, Shc, and Grb2 simultaneously docked to EGFR at pY992, pY1148, and pY1068, respectively.

The fundamental concept in this approach is that coarse-grained molecular docking is similar to assembling a jigsaw puzzle. The docking software, PatchDock [44], is based on shape complementary principles and shape matching algorithms. We used PatchDock to estimate the capability for combinations of four different adaptor proteins to bind to both tails in an asymmetric EGFR dimer (Figure 1A). We focused on Grb2, Shc, PLCγ1 and Stat5, beginning with homology modeling and protein structure prediction methods to arrive at a range of potential 3D structures; details of these approaches are found in Material and Methods and illustrated in Figure 1B. The asymmetric model of the EGFR kinase domain dimer was based upon the crystal structure solved by Zhang and colleagues [45]. In this model, the distal surface of the C lobe of one kinase domain interacts with the N lobe of the other kinase domain in the dimer. In this orientation, only one kinase in the pair is activated, leading to transphosphorylation of the other C-terminal tail. However, if the EGFR juxtamembrane domain is flexible, it is possible that the two kinase domains in the dimer can switch positions dynamically to result in activation and phosphorylation of both receptor tails [45]. In the simulations described below, we assume participation of both receptor tails in a dimer based upon this flexibility theory. Structural details of the C-terminal region of the tail are missing, since crystals were formed from recombinant proteins truncated after the kinase domain. However, the final 200 amino acid stretch of the EGFR tail appears to lack a predicted secondary structure (Fig 1A) and is likely to be highly flexible. This conclusion is consistent with FRET studies showing that activation leads to separation of the tail from the tyrosine kinase domain giving a more extended molecule [46].

The docking model is based the assumptions in Figure 1C (adapted from [15]). Only four of EGFR's phosphotyrosine sites are shown, along with their known interactions with Grb2, Stat5, Shc and PLCγ1. The predicted structures of Grb2, Shc, Stat5, PLCγ1 and the EGFR tail were input to PatchDock, along with specifications of their domains for docking to defined tyrosine residues in the EGFR tail. As expected, many solutions result from this type of simulation. Figure 1D-G show examples of the docking configurations predicted by PatchDock, with additional examples illustrated in the Supplemental Data (Additional file 1). We focused on situations that permitted multiple adaptors to dock *simultaneously*, as well as situations that *excluded *one or more adaptors from docking on an occupied tail. Based upon these results, we developed Table 1 as a foundation for docking rules in the agent-based model. For example, the coarse-grained docking model suggests that the docking of the relatively large PLCγ1 molecule (molecular weight of 145 kDa) to either Y992 or Y1173 prevents another PLCγ1 docking at the remaining unoccupied site. Stat5, PLCγ1, and Grb2 can dock to pY992, pY1173 and pY1068 sites of an EGFR tail, respectively, and the coarse grain model suggests any two or all of them can feasibly dock to the tail at the same time ("sharing"; Table 1, plus symbols in the first row). Note that we make no claim that the coarse-grain method accurately reflects the orientation of proteins bound in the complex. Instead, our goal was to ask if there appeared to be sufficient space to accommodate more than one protein on a given EGFR tail. Thus, our coarse-grained molecular docking model predicts combinations of the four adaptor proteins that might *reasonably be expected *to bind a single cytoplasmic domain of EGFR. Note also that, because the C-terminal tails are highly flexible, no additional hindrances were predicted based upon similar simulations incorporating both tails of an asymmetric dimer (not shown).

**Table 1:** Docking rules for adaptors on EGFR cytoplasmic tails, as established by coarse-grained molecular docking modeling simulations

	Stat5 (PY992)	PLCγ1 (PY992)	PLCγ1 (PY1173)	Shc (PY1148)	Shc (PY1173)	Grb2 (PY1068)
Stat5 (PY992)			**+**			**+**
				**+**	**+**	**+**
PLCγ1 (PY992)				**+**	**+**	**+**
PLCγ1 (PY1173)	**+**					**+**
Shc (PY1148)	**+**				**+**	**+**
		**+**			**+**	**+**
Shc (PY1173)	**+**			**+**		**+**
		**+**		**+**		**+**
Grb2 (PY1068)	**+**			**+**	**+**	
		**+**		**+**	**+**	
	**+**		**+**			

Plus (+) symbols on the same row indicate where docking simulations support the possibility for this combination of adaptors to simultaneously bind to phosphorylated tyrosine residues on the same EGFR tail. For example, from the first row, when Stat5 is bound to pY992, either one or both of PLCγ1 (pY1173) and Grb2 (pY1068) can bind simultaneously. Or, any or all of Shc (pY1148), Shc (pY1173), and Grb2 (pY1068) can bind simultaneously with Stat5 (pY992).

### Simulation of EGFR phosphorylation/dephosphorylation kinetics

**Figure 2:**
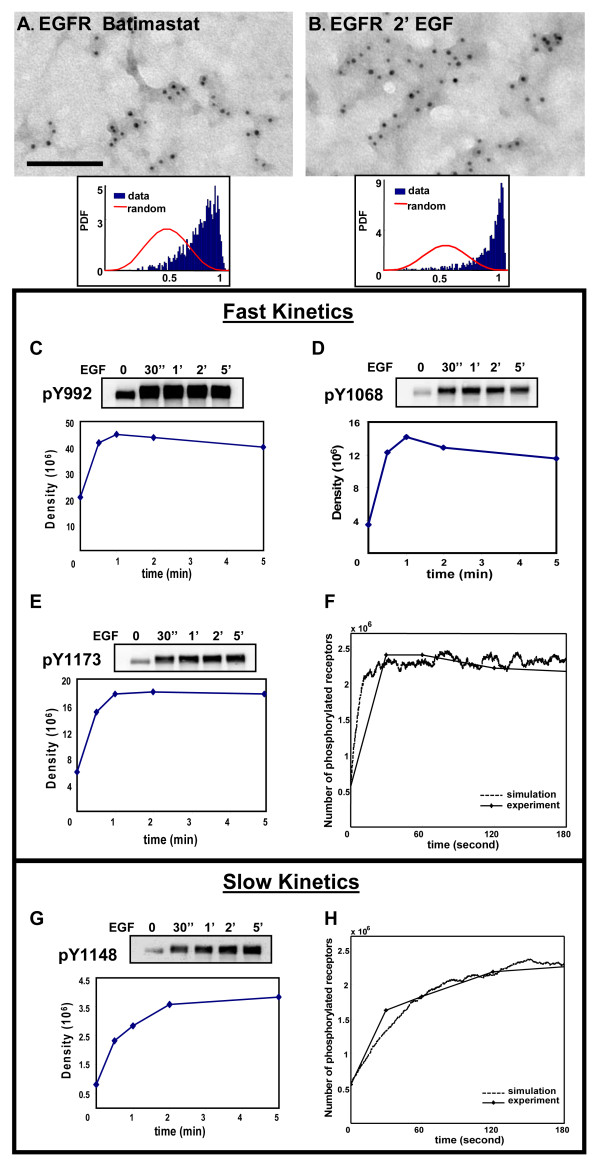
**Analysis and simulation of EGFR tyrosine residues phosphorylation kinetics**. A431 cells were serum-starved and treated with batimastat for "resting" condition shown in (A), or treated thereafter for 2 min with 20 nM EGF (B). "Rip-Flips" were prepared and membranes immunogold-labeled with anti-EGFR antibodies. Inset in both (A) and (B) confirms EGFR clustering by Hospkins test. Bars, 0.1 μm. Western blotting method was used to analyze phosphorylation kinetics of EGFR tyrosine residues (**C**) Y992, (**D**) Y1068, (**E**) Y1173, and (**G**) Y1148. Bands were quantified by densitometry and plotted as density of the bands. (**F**, **H**) Results of simulations (dashed lines) agree well with the "fast" kinetics and "slow" kinetics data (solid lines), using parameter values estimated by fitting to the data.

In preparation for spatial simulations, we characterized receptor distributions in EGF-treated cells and determined the phosphorylation/dephosphorylation kinetics for each of the four tyrosine residues. Our study is based upon data from A431 breast cancer cell line. Based upon our own flow cytometry measurements of ErbB family receptor levels [41], this line expresses over 4 million EGFRs and very little of other ErbB family members. Electron microscopy (EM) images in Figure 2A report the nanoscale spatial distribution of EGFR on membrane sheets produced using the "rip-flip" technique [47] from A431 cells after 2 hrs of serum starvation and batimastat treatment to prevent stimulation by serum-derived or shedding of EGFR ligands. Resting EGFR are distributed in small clusters (Figure 2A), typically with an increase in cluster size following addition of EGF (Figure 2B). We applied the Hopkins test to confirm that EGFR distributions are significantly non-random. The Hopkins spatial statistical test has been extensively used by our group [33,48]; a right shift of the data is interpreted as evidence for clustering (Figure 2A-B, inset). Western blots in Figures 2C-E,G compare the kinetics of phosphorylation on tyrosine residues 992, 1068, 1148, and 1173 in EGF-treated A431 cells, for which reliable phospho-specific commercial antibodies are available. Phosphorylation of Y992, Y1068 and Y1173 all peaked at 30-60 seconds; these three residues are grouped into one category that is considered to have "fast" kinetics (Figure 2F, solid line). Phosphorylation of Y1148 peaked at much later time (~5 minutes) and is considered to be in a different category with "slow" kinetics (Figure 2G, solid line). These data were digitized by densitometric scanning and quantitative results plotted as unitless values below each blot in the series. Note that resting A431 cells have detectable phosphorylation at all 4 sites, that increases by 2 to 4-fold at the peak values after EGF stimulation.

Phosphorylation and dephosphorylation rate constants were estimated by fitting to this experimental data, using the time course of pY992 to represent the "fast" category and the time course of pY1148 to represent the "slow" category. Based upon stochastic simulations, we estimate that exposure to 20 nM EGF should result in 60% of total EGFR in A431 cells within ligand-bound dimers at steady state. We assumed that ligand-bound dimers have 2-fold higher probability of tyrosine phosphorylation ([39], Table 2) than do ligand-less dimers. Extending our earlier work ([33] and that of [49], we assumed that 14% of *unoccupied *EGFR were in transient dimers and that the observed receptor phosphorylation at resting state (Figure 2C,D,E,G, zero time) is contributed by these predimers. Therefore, at t = 0, both categories are assumed to begin with 14% of receptors phosphorylated (560 000 receptors); at the peaks for both categories, 60% of the receptors are phosphorylated (2,400 000). The number of phosphorylated receptors at other time points were derived based on the density value at these time points compared to the peak value. Using this method, we arrive at <560,000 PY992 at time zero, with values of 2,394,240, 2,400,000, 2,215,200, and 2,065,440 PY992 at t = 30, 60, 120, and 180 seconds, respectively.

**Table 2:** Estimated rate constants for EGFR phosphorylation/dephosphorylation and adaptor recruitment

Parameter description	Value
Receptor tyrosine phosphorylation rates	
Residue 992	0.055 *(nM × s)*^-1^
Residue 1068	0.055 *(nM × s)*^-1^
Residue 1148	0.0063 *(nM × s)*^-1^
Residue 1173	0.055 *(nM × s)*^-1^
Multiplier when a receptor is phosphorylated.	3
Multiplier when a receptor is ligand-bound.	2
	
Receptor tyrosine dephosphorylation rates	
Residue 992	0.013/s
Residue 1068	0.013/s
Residue 1148	0.0014/s
Residue 1173	0.013/s
Multiplier when ligand is removed.	4
	
Adaptor docking rates	
For Grb2	0.0072 *(nM × s)*^-1^
For Stat5	0.0055 *(nM × s)*^-1^
For PLCγ1	0.00216 *(nM × s)*^-1^
For Shc docking to pY1148	0.00936 *(nM × s)*^-1^
For Shc docking to pY1173	0.0056 *(nM × s)*^-1^
	
Adaptor dissociation rates	
For Grb2	9.34/s
For Stat5	11.74/s
For PLCγ1	21.15/s
For Shc dissociation from pY1148	13.39/s
For Shc dissociation from pY1173	13.39/s
	
K_d _of adaptor recruitment to EGFR	
For Grb2	1.3 μM
For Stat5	2.13 μM
For PLCγ1	9.8 μM
For Shc dissociation from pY1148	1.43 μM
For Shc dissociation from pY1173	2.4 μM

We used the PottersWheel parameter fitting toolbox [50] to estimate the "fast" and "slow" kinetics of tyrosine phosphorylation. Details of this ODE-based approach are found in Computational Methods. The estimated phosphorylation and dephosphorylation rate constants of the "fast" kinetics category are 0.055 (nM×s)^-1 ^and 0.013/s, respectively. The rate constant estimated for "slow" kinetics phosphorylation is 0.0063 (nM×s)^-1^, with a dephosphorylation rate 0.0014/s (Table 2).

Stochastic simulations by SPS [33] were used to validate these estimated parameter values. Based on a value of 4 million receptors per A431 cell, the simulated cell membrane area of 0.49 μm^2 ^contained 1592 EGFR particles. To set up the initial condition with 14% EGFR predimerized in resting A431 cells, we used the receptor conformational flux model from our previous work [33]. In this model, collision between two transiently "open", dimerization-competent receptors leads to ligand-independent dimerization. We used a simulation time step of 25 μs, a random distribution of EGFR, a diffusion rate of 0.09 μm^2^/s for receptors, and 20 nM EGF. Ligand binding, dissociation, and receptor dimerization rate constants came from Shankaran's model [39] and are found in Computational Methods. As shown in Figures 2F and 2H, simulations run with the estimated parameter values for the "fast" and "slow" kinetics show close agreement with the experimental data.

### Simulation of kinetics of EGFR with its adaptor proteins

The next step was to establish reasonable reaction rate constants for simulating receptor docking and dissociation for the same four signaling proteins considered in Table 1 (Shc, PLCγ1, Stat5 and Grb2). Table 3 presents a summary of affinities reported in the literature for the binding of SH2 and PTB domains to their targets. Most studies provide only an equilibrium dissociation constant, K_d_, although single particle tracking methods were recently used to estimate the multi-state reaction rate constants between Grb2 and EGFR in A431 cell membrane preparations [32]. Our goal was to arrive at rate constants consistent with the range of reported values, but derived by parameter fitting to our own experimental data sets obtained in A431 cells.

**Table 3:** Binding constants reported in literature

Binding domain	Target	K_d_	K_on_	K_off_	Method	Reference
SHC PTB	EGFR (32 PY peptides)	<2 μM			Protein microarray & SPR^a^	[26]
	EGFR PY1148 peptide	28 nM			ITC^b^	[71]
	TRK PY490 peptide	42 nM				
SHC SH2	EGFR PY1148 peptide	NB^c^			SPR	[27]
	EGFR PY1173 peptide	65 nM				
PLCγ1 SH2	EGFR PY peptides	<2 μM			Protein microarray	[26]
PLCγ1 N+C SH2	EGFR PY992	200 nM			SPR	[28]
	EGFR PY1173	740 nM				
STAT1,3 SH2	EGFR PY peptides	>2 μM			Protein microarray	[26]
Grb2 SH2	EGFR PY1068 peptide	30 nM			SPR	[27]
	EGFR PY1086 peptide	60 nM				
	EGFRPY1148 peptide	NB				
	EGFR PY1173-7Y4	NB				
	EGFR PY992-8Y4	NB				
	EGFR phosphopeptides	<2 μM			Protein microarray	[26]
Grb2 protein	EGFR PY1068 peptide	380 nM			ITC, SPR	[31]
	EGFR PY1068 peptide	713 nM			SPR	[29]
	Activated EGFR in A431 membranes (fractional)	97-650 nM	0.016/nM/s (78%)	7.5-8.1/s (89%)	Single molecule analysis	[32]
			0.005/nM/s (21%)	1.6-2.6/s (<11%)		
			0.0022/nM/s (1%)	0.07-0.4/s (<5%)		
p85 N-SH2	PDGFR PY315 peptide	300 nM			ITC, SPR	[72]
p85 2 SH2	IRS-1 PY	0.3-3.0 nM	0.03-0.4/nM/s	0.11-0.19/s	SPR	[73]
Src SH2	(YMXM) peptides polyoma middle tumor antigen	600 nM			ITC	[74]
Lck SH2	Lck Y505 peptide	4 μM			ITC, SPR	[72]
	EGFR C-terminus	5 μM			ITC	[74]

^a^SPR = Surface Plasmon Resonance, ^b^ITC = Isothermal Titration Calorimetry, ^c^NB = No binding

**Figure 3:**
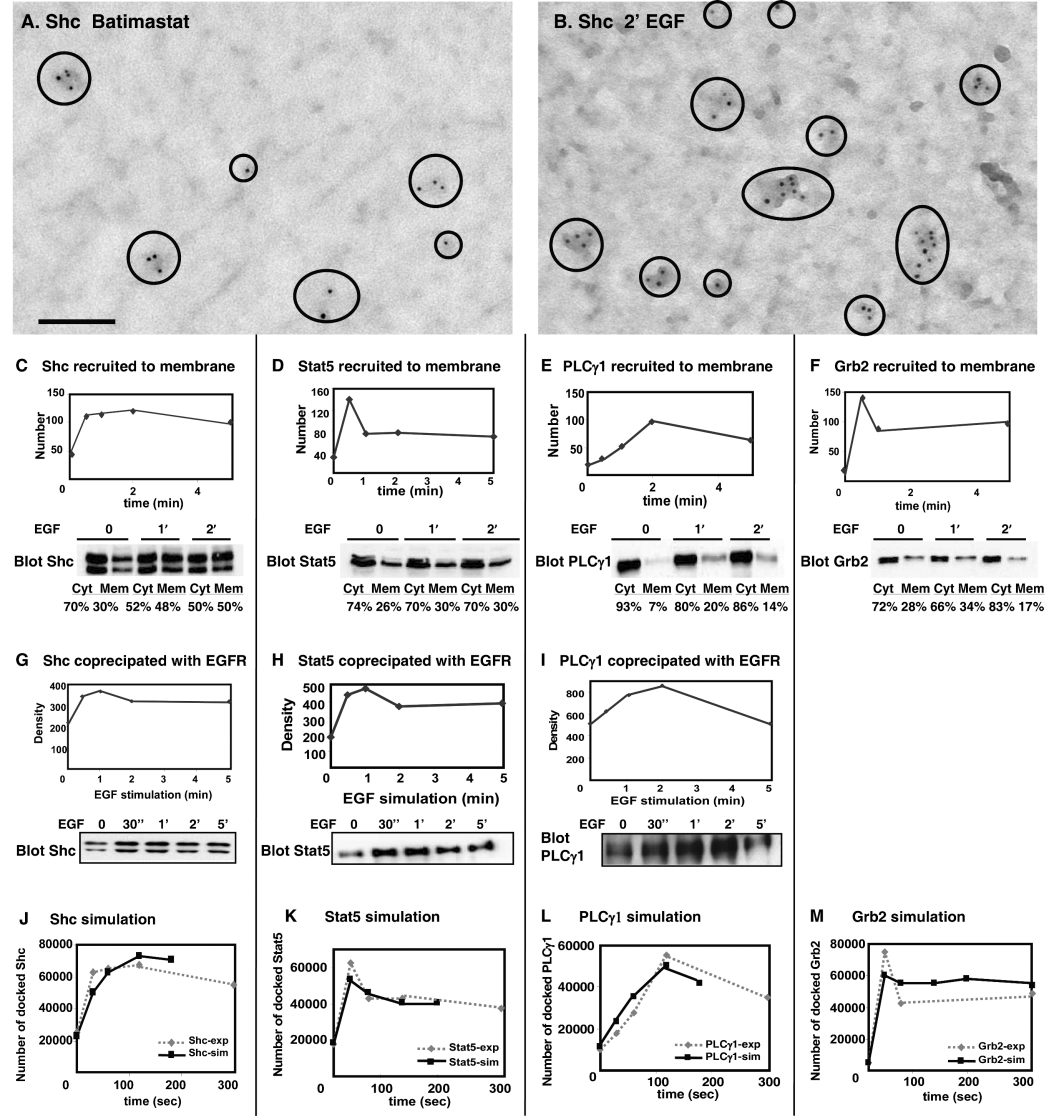
**Analysis and simulation of the reaction kinetics between the four adaptors and EGFR**. (**A-B**) Membrane sheets were prepared from serum-starved, batimastat-treated A431 cells without (A) or with EGF stimulation (**B**). Sheets were labeled with 5 nm gold reagents recognizing Shc. Circles in (**A, B**) highlight Shc label on these membranes. Bars, 0.1 μm. (**C-F**) Quantitative values of Shc, Stat5, PLCγ1, and Grb2 immunogold labeling on 3 μm^2 ^area of membrane, reported as an average of at least 10 membranes. Blots in **C-F **show results of fractionation experiments, where crude cytosol and membrane fractions were prepared, proteins separated by SDS-PAGE and membranes blotted for Shc, Stat5, PLCγ1 and Grb2. In (**G-I**), blots report co-precipitation of Shc, Stat5 and PLCγ1 with EGFR over a time course of EGF stimulation. Bands were quantified by densitometry and plotted as density of the bands. In (**J-M**), simulations of reaction kinetics between the four adaptors and EGFR using experiment-fitted values produce results (black solid line) similar to experimental data (grey dashed line).

In Figure 3, we report results of three complementary techniques to evaluate the time course and extent of recruitment of these four proteins to activated EGFR. Figure 3A-B demonstrate the use of membrane "rip-flips" and immunoelectron microscopy to document the recruitment of Shc to plasma membranes of EGF-treated A431 cells. In this assay, fixed membranes are incubated with saturating amounts of anti-Shc primary antibodies, followed by labeling with secondary antibodies conjugated to electron-dense 5 nm gold particles. Results of Shc recruitment over a time course of EGF stimulation are reported in the plot in Figure 3C (top), providing the average number of Shc in a 3 μm^2 ^area of membrane before correction for an estimated labeling efficiency of 70%. With an approximate surface area of 1256 sq microns for the whole cell, this translates to about 69,000 Shc molecules associated with A431 membranes at 2 min of EGF treatment after accounting for underlabelling. The kinetics of Shc recruitment to the membrane compare favorably with the increase in Shc that coprecipitated with EGFR over the same time course (Figure 3G). Finally, we used cell fractionation methods to estimate the fraction of Shc molecules in both membrane and cytoplasmic pools (Figure 3C, bottom). Extrapolating from the value of 69,000 Shc on A431 cell membranes at 2 min of EGF, with another 50% in the cytosol, we arrive at an estimate of 138,000 total Shc in A431 cells. This process was repeated for the other 3 proteins (Figures 3D-I), generating estimated values of 141,000 Grb2, 148,000 Stat5 and 387,000 PLCγ1 per cell.

**Figure 5:**
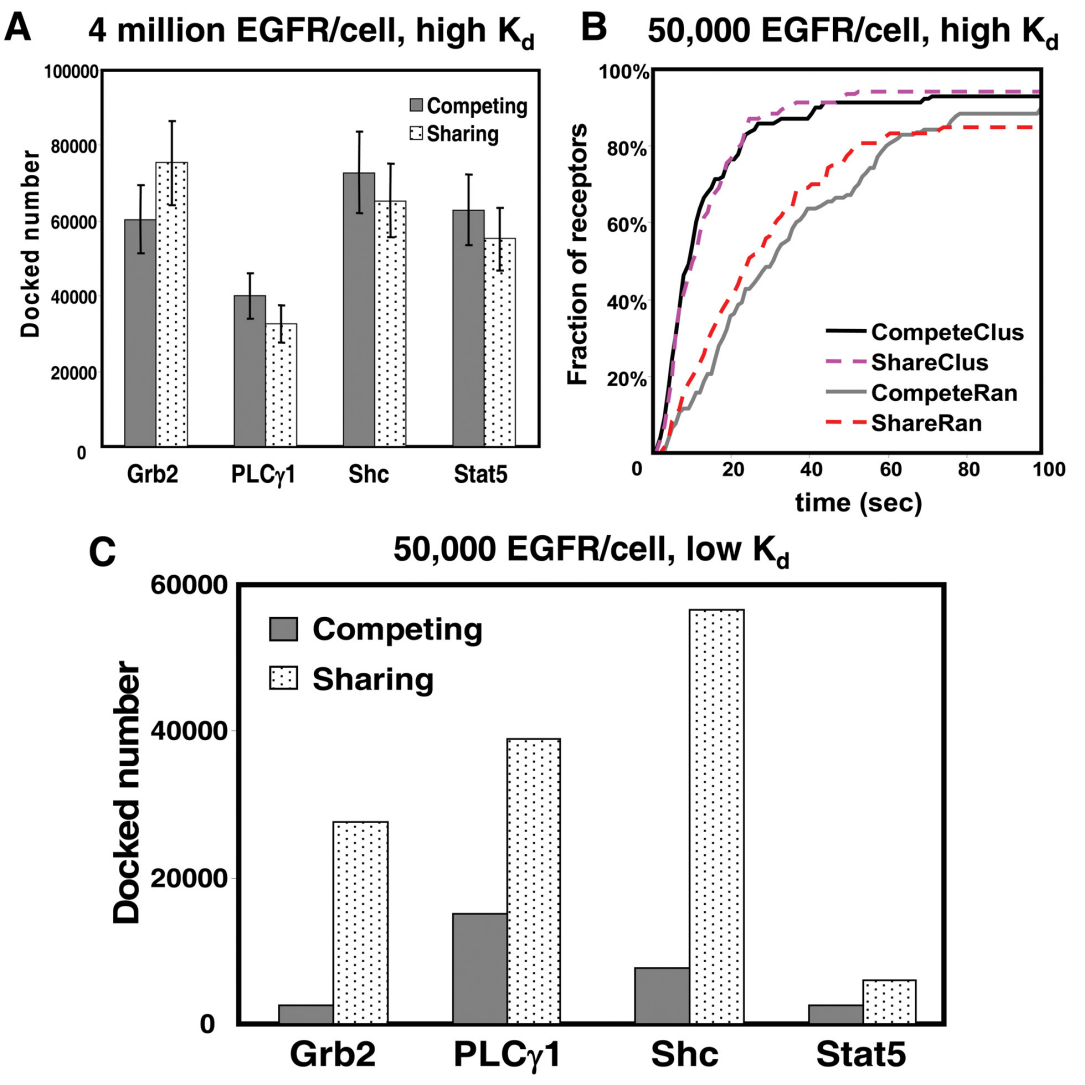
**Comparison of the sharing and competing docking models**. Simulations in (**A-B**) used the parameter values fitted to A431 cells data, while simulations in **(C) **used Kholodenko's parameter values [36]. (A) Results of simulations show similar number of adaptors docked to EGFR at steady state, when receptors are overexpressed and clustered. (B) Results of simulations for sharing and competing models at steady state, when receptors are at normal expression levels (50,000 receptors/cell) and either clustered or random. Receptor clustering increases the efficiency of adaptor retainment to EGFR, but sharing does not contribute further efficiency. (**C**) Results show that use of slow dissociation rates produces a dramatic increase in the shared docking of adaptors on EGFR tails, simulated for 50,000 clustered receptors in the spatial-stochastic model.

Rate constants were next derived by parameter fitting using the Potters Wheel toolbox, building on the parameters established for phosphorylation kinetics in Figure 2. Because this is an ODE-based approach that assumes a well-mixed chemical system, the docking rate constants were multiplied by a scaling factor (*f *in equation 3) prior to testing for fitness in our agent-based, spatially heterogeneous model. In simulations, the extracellular domain of the model was populated with 20 nM EGF, the simulated membrane expressed 1592 EGFR, and the intracellular domain contained either 56 Grb2, 55 Shc, 59 Stat5 or 154 PLCγ1. Simulations were run for each of the four adaptors individually and parameters adjusted to match the experimental data (Figure 3J-M). The docking and dissociation rate constants arrived at for each protein using this computational method are reported in Table 2. Note that these values are similar to those predicted by single particle methods [32] but that the dissociation rate constants are significantly faster than previously used in deterministic models. As discussed in the context of Figure 5C, this has a large impact on the spatial simulation outcome and experimental verification is a priority for our future work.

### Effect of receptor clustering on efficiency of signal transduction

**Figure 4:**
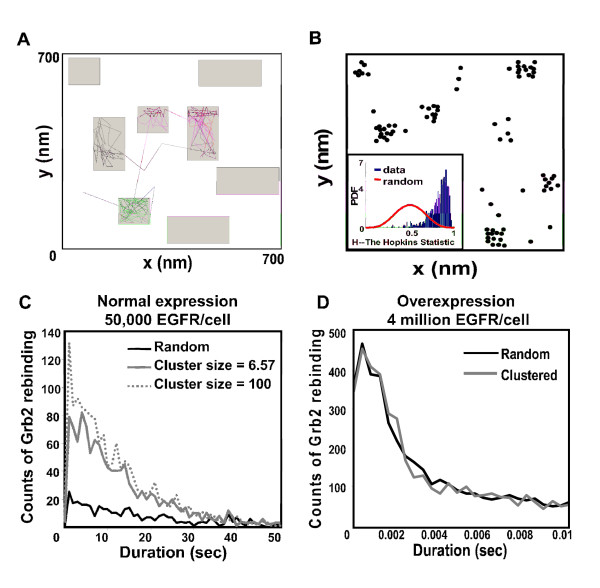
**Effect of receptor clustering and density on efficiency of adaptor containment near the membrane**. (**A**) Illustration of particles diffusing in and out of preferred domains or islands. Colored traces show 10 second trajectories of 3 diffusing EGFR particles. Receptors have greater probability to enter preferred domains, where they diffuse 3x slower. When outside of domains, particle diffusion is unconstrained. (**B**) Particles are clustered at every time step when diffusion is governed *in silico *by the domain approach. Inset shows results of Hopkins test, confirming clustering is significant. Plots in (**C**) show that receptor clustering increases the efficiency of Grb2 retainment to EGFR at normal levels of expression (50,000/cell). The frequency of Grb2 docking to another receptor after dissociating from a previous binding event is low when receptors are in the randomly distributed (black line). Increasing receptor cluster size increases the efficiency of adaptor containment near the membrane (grey solid line, 6.57 receptors/cluster; grey dashed line, 100 receptors/cluster). In contrast, if EGFR is overexpressed the plots (**D**) show no difference in Grb2 rebinding.

To this point, simulations were based on randomly distributed membrane receptors, with a uniform diffusion rate of 0.09 μm^2^/s. However, as shown in Figure 2A-B, EGFRs are highly clustered on the A431 cell surface. In our prior work, we developed a membrane compartmentalization approach (Protein Islands) to simulate receptor clustering in membranes [33]. The simulated membrane is populated with subdomains or islands ranging from in size from 50 nm^2 ^to 300 nm^2 ^(Figure 4A); the area covered by these subdomains is determined from EM images. Receptor agents are assigned with a higher probability to enter (0.9992) than exit (probability = 0.0008) the islands. Agents also slow down by a third (diffusion coefficient = 0.03 μm^2^/s) when they diffuse within the islands and resume fast diffusion (0.09 μm^2^/s) when they exit the islands [33]. To confirm that the simulated data produce non-random distributions, we utilized the Hopkins test for clustering. Importantly, results show good agreement between both simulated data (inset of Figure 4B) and real images (Figure 2, insets).

Using this approach, we compared the efficiency of adaptors that are retained on EGFR in the randomly distributed and clustered topography. Under both conditions, simulations included 20nM EGF, 100 EGFR and 280 Grb2 (equivalent to approximately 50,000 EGFR and 141,000 Grb2 per cell). In the clustered condition, we compared cluster sizes of 6.57 and 100 receptors per cluster. Figure 4C shows histograms plotting the number of events where Grb2 docked to another receptor within a 50 second interval after dissociating from a previous binding event, with comparisons in the three spatial environments. Receptor clusters of 100 increase the efficiency of Grb2 rebinding to a second EGFR by 6 fold, compared to randomly distributed receptors at this normal expression level. Overall efficiency of receptor coupling during the first 60 seconds is markedly higher in the clustered state (Figure 5B). This is consistent with the concept that an adaptor dissociating from a receptor has two possible outcomes: it can diffuse back into the cytosol or collide with the membrane. According to this scenario, receptor clustering creates a local density, increasing the likelihood that the adaptor will collide productively with a nearby membrane receptor.

Results in Figure 4D show that the increased efficiency of coupling contributed by receptor clustering would be obscured if overexpression creates a high *overall *density in the membrane. When using conditions applicable to the highly aggressive A431 cancer cell line (4 million receptors), plots for docking efficiency are essentially identical in the random and clustered state. This supports the hypothesis developed from our previous work that both receptor density and membrane spatial organization may be important factors in the carcinogenesis process [33].

### Sharing docking model may suggest more efficient and diverse signaling output

In Figure 5A-B, we conclude by examining the frequency of simultaneous adaptor recruitment to activated EGFR under conditions of normal (50,000) and high (4,000,000) levels of receptor expression. Rules for permitted combinations of adaptors and signaling molecules on a single phosphorylated receptor tail were based upon our coarse-grained docking approach (Figure 1, Table 1). This work builds on that described in previous figures, including rate constants for docking and dissociation, as well as phosphorylation/dephosphorylation. The cytosolic simulation space was populated with identical numbers of the four signaling proteins (56 Grb2, 55 Shc, 59 Stat5, and 154 PLCγ1), while receptors varied from 20 (representing 50,000 receptors in the whole cell) to 1592 (4 million receptors/cell, as in A431 cells) for the case of random topography. To simulate clustered topography for the normal case of 50,000 receptors/cell, the simulation space was expanded 5-fold, with a corresponding increase in receptors and adaptors to match the new cellular volume representation. For each level of receptor, we compared results based upon the "sharing" docking model with results generated using a "competing" docking model. In the latter case, occupancy of a receptor tail at any given time step excluded another signaling protein or adaptor from binding to the same tail.

Simulation results in Figure 5A-B are intriguing in that they predict that the capability for "shared" docking does not significantly affect overall recruitment of adaptors, even in simulations using high density of receptors (either through clustering or overexpression). This result is intuitive in the case of EGFR overexpression, where the number of receptors is five times that the total number of the four adaptors (Figure 5A). This result was initially unexpected in the case where receptors fall well below the level of adaptors (50,000 receptors/cell, Figure 5B). However, this is explained by the prediction that the shared docking model should be profoundly dependent upon the rate constants applied in the simulation for *dissociation *of proteins from EGFR. This is illustrated in Figure 5C, where we substituted our docking and dissociation rate constants for Grb2, Shc and PLCγ1 with those of Kholodenko's model [36]. For Stat5, we substituted the K_d _estimated by Shao et al for docking of Stat3 to the phosphododecapeptide Y1068 [51]. The simulations had 20 receptors in clustered topography in the simulated space (50,000 receptors per cell), 20nM EGF and the same numbers of the four adaptors as before. Figure 5C shows that use of these slower dissociation rate constants results in very large differences in total numbers of adaptors recruited using the two docking models. There are up to 7.5-fold increases in adaptors docked to EGFR at steady state using the sharing model, compared to the competitive model. If the dissociation rate of proteins bound to EGFR is slow, the formation of large complexes on the EGFR tail is likely to be a frequent event.

## Discussion

In this work, we apply agent-based, stochastic model to investigate mechanisms of adaptor proteins recruitment to EGFR as functions of time, receptor conformation, density and spatial distribution. Unique features include the inclusion of docking rules to consider the problem of combinatorial complexity and the consideration of cell membrane heterogeneity. Parameter values used in the model were estimated by fitting to our own western blotting and immunoelectron microscopy data from A431 cells, which provide evidence of distinct phosphorylation kinetics for different EGFR tyrosine residues and distinct behavior for adaptors recruited to phosphorylated receptors. Previous models for studying ErbB receptor signaling represented all tyrosine residues as a single 'virtual phosphorylation site' and assumed competition among cytoplasmic adaptors for receptor binding [34,36-39]. The rule-based model of Blinov et al [35] can account more fully for potential molecular diversity; however, like others it is a differential equation model that cannot well describe cell surface heterogeneities, such as microdomains [52] or anomalous diffusion of surface receptors [53].

Our experimental data for resting cells were derived from serum-starved and batimastat-treated A431 cells, conditions that control for serum factors or autocrine stimulation (by shedding of EGFR ligands). It is notable that, at t = 0, there are already detectable levels of EGFR tyrosine phosphorylation and adaptors docked to EGFR (Figure 2). We hypothesize that these are contributed from the ligand-independent EGFR dimers as observed by many groups [33,40,49,54-57]. Therefore, we made some assumptions when quantifying the western blotting data of receptor tyrosine residues phosphorylation: First, the observed phosphorylation at resting is entirely contributed from transient dimers formed by encounters between conformationally fluxing receptors. This process is density dependent and estimates of these "constitutive" and unstable dimers in A431 cells range from 5% [58] to 14% [49]; we use the latter value for our simulations. Second, we assume that the rise in tyrosine phosphorylation is equivalent in both receptors within the dimers that form (60% participation at peak values after treatment with 20 nM EGF). This may be an overestimate, since it is unknown whether both tails in the asymmetric dimer [45] are equally capable of phosphorylation. Indeed, there is evidence that receptor phosphorylation achieves values of only 10-35% in mammary epithelial cells [59], which would be consistent with unequal transphosphorylation by the two kinases in an EGFR homodimer. We also ignored internalization of receptors in the present work, although we acknowledge that this may be a component of the "fast" and "slow" kinetics for the four tyrosine residues whose phosphorylation kinetics we studied. The deterministic models of Wiley [38,60] and Kholodenko [34] have considered the importance of EGFR endocytosis, particularly in the contexts of dimer composition and EGFR mutations, and we anticipate adding this feature to the SPS simulation model as we learn additional molecular details about the kinetics and docking characteristics of AP2 and clathrin recruitment.

Based upon parameter fitting, we estimated that the association (k_on_) and dissociation (k_off_) rate constants between Grb2 and EGFR are 0.0072 (nM × s)^-1 ^and 9.34/s, respectively. Remarkably, these values are very close to the reported rate constants measured by single-molecule analysis (k_on _= 0.0022-0.016 (nM × s)^-1^; k_off _= 7.5-8.1/s) in the same cell line [32]. We assumed that Shc PDB domain is the predominant means for recruitment to the EGFR, with pY1148 as a preferred site and pY1173 as the secondary binding site [61,62]. We arrived at estimated parameter values for Shc to these two sites, with a higher k_on _for pY1148 (= 0.00936 (nM × s)^-1^) than pY1173 (= 0.0056 (nM × s)^-1^) and the same k_off _(13.39/s) for both. The estimated K_d _values of adaptor interactions with EGFR in our studies are of the order of 1 μM (Table 2), and these values are close to those in recent reports [26,32]. This relatively low affinity is consistent with the estimates that k_off _is large, and k_on _is small, such that adaptors can form complexes with EGFR and still can rapidly dissociate to limit signaling duration (Grb2/Shc) or propagate signals (Stat5, PLCγ). In this context, receptor clustering would promote rebinding to another active EGFR and provide a mechanism to enhance signaling efficiency despite high dissociation rates (Figure 4 and 5) A high overall density of receptors, typical of cancer cells that overexpress EGFR, also creates conditions of enhanced signaling efficiency.

It is important to note that, although simulations with these values agree well with the experimental observations, they are up to 100 fold higher (ie., lower affinity) than K_d _estimates based upon the binding properties of recombinant SH2 domain and PDB domain to target phosphopeptides (see Table 3 and references therein). This is potentially due to "multi-state" interactions between intact receptors and adaptors, as suggested for EGFR-Grb2 interactions measured by single-molecule analysis [32]. Thus, we caution that the estimated reaction rate constants in this work served solely as references for stochastic modeling. More precise rate constants need to be determined based upon novel experimental techniques for measuring binding between intact molecules in a biological context. As shown in Figure 5, the reaction rates have a large impact on the potential for assembly of large complexes on the EGFR tail. If dissociation rates are fast, combinatorial complexity will be low. If dissociation rates are slow, there is much greater chance for a single EGFR tail to recruit multiple signaling components where permitted by the constraints of steric hindrance.

To our knowledge, this is the first attempt to try to establish rules for simultaneous or competitive docking of adaptors to phosphorylated receptor tails, followed by application of these rules in simulations of single molecule behavior. To accomplish this, we relied upon coarse-grained docking predictions. These methods incorporate information about known or predicted structural domains, using homology and protein structure prediction methods. Domains were linked together by an approach previously applied to modeling of large RNA [63]. Each coarse-grained protein structure was then docked, alone or in combination with the other adaptors, to a flexible C-terminus tail of EGFR based upon their shape complementary using PatchDock. The predicted rules are summarized in Table 1. This inexact approach provided several useful exclusionary rules for testing by simulation. For example, PLCγ1 appears to be sufficiently large that two cannot dock to a single receptor tail, despite the availability of 2 distinct binding sites. PLCγ1 docked at pY1173 likely also prevents Shc docking on the same receptor. We emphasize that the limitations of the coarse grain approach raise many uncertainties about these rules. In addition, there are many unknowns related to the asymmetric model of EGFR dimer, particularly the assumption that both kinases in the dimer become activated by conformational switching [45]. All of these assumptions point to the need for additional experimental validation in order to develop more accurate models.

## Conclusions

Mathematical modeling is most useful when it suggests new priorities for coupling of experimentation and simulation. From this work, we identify several areas for future development. First, we seek new insight into the mechanisms that drive receptor clustering and microdomain formation. Our simulation results suggest that, when clustering is introduced, adaptors are retained more readily at the plasma membrane. We predict that this efficiency would increase as the receptor cluster size increases (Figure 4C and 5B). At "normal" receptor expression levels, receptor clustering should create high local density and enhance the probability that dissociated adaptor proteins collide quickly with another receptor. However, simulations also suggest that overexpression of receptors have the same effect as receptor clustering, presumably because an overall high density of receptors improves the chances for dissociating adaptors to rebind. This supports the prediction from our previous work that both receptor density and membrane spatial organization contribute to the carcinogenesis process [33]. Second, the coarse-grained docking results suggest that simultaneous adaptor docking to a single receptor should be possible but that the significance of this possibility is highly dependent upon the stability of receptor-adaptor complexes. Related to this is the need for accurate rate constants for receptor-adaptor complexes. We propose that this effort should take precedence over attempts to confirm the coarse grain docking results, since the problem of combinatorial complexity appears to be minimized if the lifetime of receptor-adaptor binding is short. The SPS platform is applicable to modeling these early signaling events and offers capabilities for extending cascades through the cytoplasm and nucleus. It should also be readily adaptable to other protein assembly problems in cells that rely on diffusion and conformational switches. It can explicitly consider aspects of membrane heterogeneity, combinatorial complexity and hierarchy of adaptor binding.

## Methods

### Computational Methods

#### Coarse-grained Protein Modeling

Our primary goal was to 1) approximate the *size and general shape *of the EGFR tail and four of its docking partners and 2) use this information to arrive at *reasonable predictions *of combinations that might be accommodated on the EGFR tail simultaneously. The first step was to account for known domain structures within each protein, using SWISS-MODEL for automated homology-based modeling. This approach relies on sequence relationships of protein domains with one or more of known structure. Core models for individual domains (SH2, PTB, PH, etc) were based upon structural templates [64]. Next, protein prediction methods were used to build regions of the structure not available from the templates. These methods combine machine learning methods, evolutionary information in the form of profiles, fragment libraries extracted from the Protein Data Bank (PDB) [65], and energy functions to predict protein structural features. The protein structure predictors used in this work were 3Dpro from Scratch [66], MaxSprout [67] and I-TASSER [68]. 3Dpro predicts protein tertiary structure and outputs PDB files as a Carbon Alpha trace. Maxsprout was used to add backbone and side chain coordinates to obtain an all-atom model. I-TASSER combines both template-based and template-free modeling approaches. All the servers used in this work have been evaluated by the CASP (Critical Assessment of Structure Prediction) experiment [69]. The overall protein structure was then generated by linking these models of sub-domains together by the software, Insight II http://accelrys.com/products/insight/, using an approach similar to that of [63] for modeling large RNA 3D structure. PatchDock [44] was then used to simulate docking of two or more structures. No claim is made regarding the accuracy of these predicted structures.

The PDB files of all the predicted protein structures are available upon request. Brief strategies for each structure follow: 1) For the EGFR tail, the target sequence (L1001 to A1210) was imported into SWISS-MODEL and the First Approach Mode was performed without preselected template files. The resulting 3D model structure contains one domain (A1118 to F1176) built with 1F4H as template structure (37% identities) and has missing sequences from L1001 to P1117 and from F1177 to A1210. These two sequences, with additional amino acids for linking (L1001 to V1133 and L1167 to A1210), were then modeled by 3Dpro and MaxSprout and were linked to the domain obtained from SWISS-MODEL by Insight II. The full-length c-terminal of EGFR was then linked to the asymmetric model of the kinase domains of EGFR dimer by Insight II. 2) Shc is a 583-amino acid protein, and the crystal structure of two domains of Shc (M111 to R317 and Q482 to L583) has been solved (1MIL and 1N3H). The protein structure prediction of the two missing sequences was performed with 3Dpro and Maxsprout. Insight II was then used to link these two models with 1MIL and 1N3H together. 3) Stat5 is 794 amino acid residues long and majority of its crystal structure has been solved (1Y1U: S138 to A690). The missing sequences with additional amino acids for linking, from M1 to E150 and from K681 to S794, were modeled by the protein structure predictor, 3Dpro and MaxSprout. The resulting model structures were then linked to 1Y1U by Insight II. 4) PLCγ1 is composed of 1290 amino acids and the crystal structure of its SH3 domain has been solved (1HSQ: 790 to 851). I-TASSER was used to model the two missing sequences (M1 to F800 and F841 to L1290), and the resulting model structures were linked to 1HSQ by Insight II. Grb2 has most of its crystal structure solved with one small missing sequence from L28 to D33. The full Grb2 was modeled by the first approach mode of SWISS-MODEL and the template was the known structure of Grb2 itself (1GRI).

#### Parameter-fitting

We used a Matlab parameter-fitting toolbox, PottersWheel [50], which provides interactive modeling including multi-experiment fitting with highly optimized model integration. An ODE (ordinary differential equations) model was first derived to represent features of the EGFR network. Experimental data was then imported and fit to the data by automatically or manually adjusting model parameters to optimize matching between model trajectory and experimental data points [50]. This strategy was used to determine the phosphorylation rate constant and the dephosphorylation rate constant for tyrosine residues 992, 1068, 1148 and 1173), using the following ODE series:

d(L)/dt = -1*k_1_*R*L + k_2_*RL;

d(R)/dt = -1*k_1_*R*L + k_2_*RL -2*k_3_*R*R + 2*5*k_4_*RR - k_3_*RL*R + k_4_*RLR;

d(RL)/dt = -1*k_3_*RL*R + k_4_*RLR - 2*k_3_*RL*RL + 2*k_4_*RLRL + k_1_*R*L - k_2_*RL;

d(RR)/dt = k_3_*R*R - 5*k_4_*RR - k_1_*RR*L + k_2_*RLR - k_5_*RR + 4*k_6_*pRR;

d(RLR)/dt = k_3_*RL*R - k_4_*RLR - k_1_*RLR*L + k_2_*RLRL - 2*k_5_*RLR + k_6_*pRLR;

d(RLRL)/dt = k_3_*RL*RL - k_4_*RLRL + k_1_*RLR*L - k_2_*RLRL -2*k_5_*RLRL + k_6_*pRLRL;

d(pRR)/dt = k_5_*RR - 4*k_6_*pRR - 3*k_5_*pRR + 4*k_6_*pRpR;

d(pRLR)/dt = k_5_*RLR - k_6_*pRLR - 2*3*k_5_*pRLR + k_6_*pRLpR;

d(pRLRL)/dt = 2*k_5_*RLRL - k_6_*pRLRL - 3*2*k_5_*pRLRL + k_6_*pRLpRL;

d(pRpR)/dt = 3*k_5_*pRR - 4*k_6_*pRpR;

d(pRLpR)/dt = 2*3*k_5_*pRLR - k_6_*pRLpR;

d(pRLpRL)/dt = 3*2*k_5_*pRLRL - k_6_*pRLpRL;

where k_1 _is the ligand binding rate constant (= 0.00000331 (# × sec/simspace)^-1^, fixed), k_2 _is the ligand dissociation rate constant (= 0.004/s, fixed), k_3 _is the dimerization rate constant (= 0.014 (# × sec/simspace)^-1^, fixed), k_4 _is the dimer dissociation rate constant (= 0.01/s, fixed), k_5 _is the phosphorylation rate constant (= 0.01 (# × sec/simspace)^-1 ^initially), and k_6 _is the dephosphorylation rate constant (= 0.005/s initially). L is the number of ligands in the simulated system (= 780, fixed), and R is the number of receptors in the simulated system (= 1592, fixed). LR is the number of ligand-bound monomer, RR is the number of ligand-free dimer, pRR is the number of dimers that have only one phosphorylated receptor, pRpR is the number of dimers of which both receptors are phosphorylated, and so on. The initial number of these complexes is set to be zero in the model. The rate constants of ligand binding, dissociation and receptor dimerization as well as the reaction rate multiplier came from Shankaran's model [39](Table 2). The simulated space, simspace, represents ~1/2512 of a typical epithelial cell. In the model, 1 (# × sec/simspace)^-1 ^equals to 0.72 (nM × s) ^-1 ^based on the assumption that the cytoplasmic water volume of a cell is 3 × 10^-12 ^l [36]. The ODE model was loaded into PottersWheel, and k_5 _and k_6 _of each tyrosine residue are estimated by fitting to the corresponding western blotting data (Figure 2F and 2H) both automatically and manually to avoid being trapped in the local optimum.

We also used this approach to estimate the parameters for binding of the four adaptor proteins (Shc, Grb2, Stat5, and PLCγ1) to target tyrosine residues. The ODE series for parameter-fitting was:

d(L)/dt = -1*k_1_*R*L + k_2_*RL;

d(R)/dt = -1*k_1_*R*L + k_2_*RL -2*k_3_*R*R + 2*5*k_4_*RR - k_3_*RL*R + k_4_*RLR;

d(RL)/dt = -1*k_3_*RL*R + k_4_*RLR - 2*k_3_*RL*RL + 2*k_4_*RLRL + k_1_*R*L - k_2_*RL;

d(RR)/dt = k_3_*R*R - 5*k_4_*RR - k_1_*RR*L + k_2_*RLR - k_5_*RR + 4*k_6_*pRR;

d(RLR)/dt = k_3_*RL*R - k_4_*RLR - k_1_*RLR*L + k_2_*RLRL - 2*k_5_*RLR + k_6_*pRLR;

d(RLRL)/dt = k_3_*RL*RL - k_4_*RLRL + k_1_*RLR*L - k_2_*RLRL -2*k_5_*RLRL + k_6_*pRLRL;

d(pRR)/dt = k_5_*RR - 4*k_6_*pRR - 3*k_5_*pRR + 4*k_6_*pRpR - 1*k_7_*pRR*AP + k_8_*pRRAP;

d(pRLR)/dt = k_5_*RLR - k_6_*pRLR - 2*3*k_5_*pRLR + k_6_*pRLpR - 1*k_7_*pRLR*AP + k_8_*pRLRAP;

d(pRLRL)/dt = 2*k_5_*RLRL - k_6_*pRLRL - 3*2*k_5_*pRLRL + k_6_*pRLpRL - 1*k_7_*pRLRL*AP + k_8_*pRLRLAP;

d(pRpR)/dt = 3*k_5_*pRR - 4*k_6_*pRpR - 1*k_7_*pRpR*AP + k_8_*pRpRAP;

d(pRLpR)/dt = 2*3*k_5_*pRLR - k_6_*pRLpR - 1*k_7_*pRLpR*AP + k_8_*pRLpRAP;

d(pRLpRL)/dt = 3*2*k_5_*pRLRL - k_6_*pRLpRL - 1*k_7_*pRLpRL*AP + k_8_*pRLpRLAP;

d(AP)/dt = -1*k_7_*pRR*AP + k_8_*pRRAP - 1*k_7_*pRLR*AP + k_8_*pRLRAP - 1*k_7_*pRLRL*AP + k_8_*pRLRLAP - 1*k_7_*pRpR*AP + k_8_*pRpRAP - 1*k_7_*pRLpR*AP + k_8_*pRLpRAP - 1*k_7_*pRLpRL*AP + k_8_*pRLpRLAP;

d(pRRAP)/dt = -1*k_8_*pRRAP + k_7_*pRR*AP;

d(pRLRAP)/dt = -1*k_8_*pRLRAP + k_7_*pRLR*AP;

d(pRLRLAP)/dt = -1*k_8_*pRLRLAP + k_7_*pRLRL*AP;

d(pRpRAP)/dt = -1*k_8_*pRpRAP + k_7_*pRpR*AP;

d(pRLpRAP)/dt = -1*k_8_*pRLpRAP + k_7_*pRLpR*AP;

d(pRLpRLAP)/dt = -1*k_8_*pRLpRLAP + k_7_*pRLpRL*AP;

where k_7 _is the docking rate constant (= 0.01 (# × sec/simspace)^-1 ^initially), k_8 _is the rate constant of adaptor proteins dissociated from a receptor (= 0.005/s initially), and the values of k_5 _and k_6 _of a tyrosine residue are determined from the previous fitting and are fixed here. AP is the number of adaptor proteins in the simulated space which is determined from experimental data and was estimated to be 56 for Grb2, 55 for Shc, 59 for Stat5 and 154 for PLCγ1. pRLRAP is the number of adaptor protein-bound dimer of which one receptor is ligand-bound and the other receptor is phosphorylated, and so on. The experimental data points used for parameter fitting were derived by converting the values obtained by immunoelectron microscopy for a 3 μm^2 ^area, as shown in Figure 3C-F, to corresponding values in our 0.49 μm^2 ^simulated space. The rate constants of adaptor proteins docking and dissociation are estimated by adjusting k_7 _and k_8 _to minimize the distance between the model trajectory (sum of the adaptor protein-bound dimer; i.e. 'pRRAP + pRLRAP + pRLRLAP + pRpRAP +pRLpRAP + pRLpRLAP') and the experimental data points. Rate constants derived by this method are reported in Table 2.

#### The Signaling Pathways Simulator

The agent-based, stochastic simulator was previously described [33]. In brief, the 3D simulation space is composed of an extracellular domain, plasma membrane and cytosol. In this work, ligands were simulated as a single concentration; however SPS can also treat ligands as individual species. Proteins in the 2D membrane and 3D cytosolic space are represented by sphere-like particles with a radius determined from experimental data and their coarse-grained molecular models. At each time step (typically 25 μs), these particles diffuse and have the potential to react with neighbors. SPS is designed for flexible model development and deployment by a modularized and rule-based approach. It tracks the individual reactions of multistate molecules and accommodates complex situations.

#### Modeling bio-reactions between EGFR and its adaptor proteins

Dimerization of receptors leads to kinase activation and transphosphorylation of the tyrosine residues on the cytoplasmic tails of the receptors. These phosphotyrosine resides then provide scaffold for various signaling molecules. In the model, the probabilities of phosphorylation and dephosphorylation of a tyrosine residue of EGFR are computed by the following two equations, respectively,(1)

and(2)

where *k*_*on *_is the phosphorylation rate constant, *k*_*off *_is the dephosphorylation rate constant (different tyrosine residues have different phosphorylation and dephosphorylation rates) and Δ*t *is the time step.

To model the biochemical interactions between EGFR and its adaptor proteins, the first step was to derive the probability, *p*(*,Δ*t *| *r*), that two particles that are *r *distance from each other in three dimension, become a complex after time step, Δ*t*, given that they can react with reaction rate constant, *k*_*a*_, and their diffusion coefficients are *D*. A similar scheme to that in our previous work [33] was used to derive this probability function of 3D reactions as shown below,(3)

where *k*_*a *_in this case is a diffusion-limited reaction rate constant, *V *is the volume of the spatially homogeneous chemical system of interest, and *f *is a scaling factor used to match the well-mixed chemical system to our agent-based spatially heterogeneous model. Its values are approximated by matching the simulation results with the western blotting and EM experimental data (Figure 3J to 3M) and are approximated to be in the range of 10,000 to 30,000 for our modeling of EGFR-adaptor protein bio-reactions.

The distance factor, *DistFac3D(r)*, is the probability of collision of two particles during time step,, given that they start a distance *r *apart in three dimensions. It is introduced to account for membrane heterogeneities and was derived by simulations by SPS. In the simulation, two sphere-like particles whose diameter is 10 nm are placed in three dimensions at a distance *r *apart initially. One particle is set to be stationary while the other one undergoes Brownian motion in three dimensions with *D *= 100 μm^2^/s. The value of *r *is ranged from 10 nm to 100 nm. The time step is set to be 5 ns, and the simulation is 25 μs long. For a given *r*, the simulation was run for 100 times. Another set of simulations was run with the same settings as described above except that the freely diffusing particle diffuses at *D *= 50 μm^2^/s instead. For a given *r*, the simulation was run for 100 times. If out of 100 simulations, there are *p *instances where the two particles collide, then the probability of two particles collide within 25 μs given that they start a distance *r *apart is *p/100*. Supplemental Figure 1 (see Figure S1 in Additional file 1) shows the relation of the probability of collision with the distance between the two particles at different *D *(circles: *D *= 50 μm^2^/s; stars: *D *= 100 μm^2^/s). The 3D distance factor is derived by fitting an equation to the data shown in supplemental Figure S1 (Additional file 1). A modified and normalized Popov and Agmon's function (equation (4) and (5)) fits the data well and is determined to be the 3D distance factor. Supplemental Figure S1 (Additional file 1) shows that the function fits to the probabilities of collision for 50 μm^2^/s ≤ *D *≤ 100 μm^2^/s (solid line: *D *= 50 μm^2^/s; dashed line: *D *= 100 μm^2^/s) and is good for modeling bio-reactions in the cytosol where particles usually diffuse at a diffusion coefficient within this range.(4)

where *r *is the distance between the two particles, *D *is the diffusion coefficient, *a *is the particle's diameter, is the time step, *i*, *j*, and *k *are different numbers from the set {1, 2, 3},(6)

and the's are the roots of the cubic equation(8)

where *k*_*a *_is the association rate constant and *k*_*d *_is the dissociation rate constant, neither of which plays a role in *DistFac(r) *after normalization in equation (5).

The probability of docking is then computed by the equation (3) to (9) where the docking rate constant, *k*_*a*_, was determined by parameter fitting to the experimental data (Figure 3J to 3M). Different adaptor proteins dock to tyrosine residues (binding sites) of a EGFR tail with different docking rate constants, they also dissociate from the EGFR tail with different exclusion rate constants. The probability of exclusion is converted from the exclusion rate constant, *k*_*e*_, by the equation as followed,(10)

The diameters and the diffusion coefficients of the four adaptor proteins of EGFR (parameter *a *and *D *in equations (4) to (9)) are determined as follows: the diameters of the four adaptor proteins, Grb2, Shc, PLCγ1, and Stat5, are determined from their protein modeling and are determined to be 6 nm, 11 nm, 11.5 nm, and 15.5 nm, respectively. It has been suggested that the diffusion coefficient of molecules, *D*, is related to the molecular weight, *M*, by the relation, [70]. The molecular weight of Grb2, Shc, PLCγ1, and Stat5 are 25 kDa, 50 kDa, 145 kDa, and 80 kDa, respectively http://www.ncbi.nlm.nih.gov/. Assuming the diffusion coefficient of Grb2 to be 100 μm^2^/s [32], the diffusion coefficients of Shc, PLCγ1, and Stat5 can then be derived and are approximated to be 80, 56, and 68 μm^2^/s, respectively.

### Diffusion

Diffusion of cytoplasmic proteins is simulated by Brownian motion while diffusion of receptor agents in the membrane is based upon the Constrained Brownian Motion Algorithm [33]. In CBM, overlap between two molecules is not permitted; this is the basis for modeling ligand-receptor, receptor-receptor, and receptor-adaptor protein bio-reactions. The algorithm is a modification of the Gilliespie's approach and is summarized as follows:

Assume a receptor agent (which could be in a dimer) has *m *neighbors, *n *of which are its binding partners (i.e. *m *≥ *n *≥ 0), and the probabilities of the reactions between the agent and these binding partners are *P*_*1*_, *P*_*2*_... and *P*_*n*_, respectively. Also assume that the receptor agent has *p *binding sites for adaptor proteins, and the probabilities of phosphorylation of each of these sites are *prob_phos*_*1*_, *prob_phos*_*2*_...and *prob_phos*_*p*_, respectively. Similarly, the probabilities of dephosphorylation of these sites are *prob_dephos*_*1*_, *prob_dephos*_*2*_...and *prob_dephos*_*p*_, respectively. If the binding site, *j*, has an adaptor proteins docked to it already, the adaptor protein can be excluded from the site with a probability, *prob_exclude*_*j *_(1 ≤ *j *≤ *p*). The functions in the simulator used to calculate these probabilities are defined such that *prob_phos*_*1*_, *prob_phos*_*2 *_to *prob_phos*_*p *_= 0 if the receptor agent is a monomer. Besides, *prob_dephos*_*j *_= 0 if the binding site, *j*, is not phosphorylated yet or if the site has an adaptor protein docked to it already. Moreover, *P*_*i *_= 0 for the receptor agent's neighboring adaptor protein, *i*, to dock to a binding site that is not phosphorylated or to a phosphorylated site that is already occupied. In addition, *prob_exclude*_*j *_= 0 if the site, *j*, has no adaptor protein docked to it yet. The receptor agent (which could be in a dimer) also has probabilities to bind ligands (*P*_*binding*_) or to dissociate (*P*_*dissociation*_). Thus, the probability of no reaction occurring at a time step equals to,
(11)

The probabilities of reactions are then scaled by , denoted as *S. S *is defined such that the sum of all probabilities of reactions scaled by *S *equals to (1- *P*_*NR*_), which is the probability of one reaction occurring. A random number, *X*, between 0 and 1 is generated to select an event for the receptor agent during the time step, permitting a specific bio-reaction to occur or else the agent undergoes constrained Brownian motion.

### Experimental Methods

#### Cell treatments and western blotting analysis

A431 cells were obtained from ATCC and cultured according to ATCC recommendations. Epidermal growth factor (EGF) was from Biomedical Technologies (Stoughton, MA). Prior to experimentation, cells were incubated for 3 hrs in serum-free medium with or without batimastat (a gift of P. McGuire, UNM), followed by lysis in ice cold 1% NP-40 buffer (150 mM NaCl, 50 mM Tris/HCl pH 7.2 with protease inhibitors). Protein concentrations in clarified lysates were measured using the BCA protein assay (Pierce, Rockford, IL). Supernatants were mixed with 6x sample buffer. Proteins were separated by SDS-PAGE and transferred to nitrocellulose. Membranes were blocked and sequentially probed with primary and HRP-conjugated secondary antibodies. EGFR antibodies were from Santa Cruz (La Jolla, CA) and phosphotyrosine-specific EGFR antibodies were from Cell Signaling (Beverly, MA). Shc antibodies were from BD Bioscience (Los Angeles, CA), PLCγ1 antibodies were from Millopore (Bedford, MA), and Stat5 and Grb2 antibodies were from Santa Cruz (Santa Cruz, CA). Immuno-reactive bands were detected by enhanced chemiluminescence (Pierce) and their intensity digitally analyzed following densitometry using MultiGauge software.

#### Preparation of plasma membrane sheets and gold labeling for TEM

Methods for labeling proteins membrane sheets are described in Yang et al [41]. Cells were grown on glass coverslips, treated as described in legends, then fixed in 0.5% PFA for 10 min. Coverslips were inverted onto EM grids (glow discharged, formvar and poly-L-lysine coated) and ripped to leave plasma membranes on the grid, cytoplasmic face up. After fixation (2% PFA, 20 min), grids were incubated sequentially with primary antibodies and gold-conjugated secondary antibodies. Samples were post-fixed with 2% glutaraldehyde, stained with tannic acid and examined using a Hitachi H-7500 transmission electron microscope (TEM) equipped with a 6.8 megapixel digital camera. A customized plugin for ImageJ was used to acquire positions of gold particles and clustering was analyzed using the Hopkins spatial statistic [48].

#### Cytosol/membrane fractionation

Cells were serum starved for 3 hours in the presence of batimastat, followed by EGF stimulation as indicated in legends. Reactions were halted by transfer to 4°C and rinsing with cold PBS. Cells were scraped off and briefly sonicated; intact cells and debris were sedimented by microcentrifugation (10 minutes). Supernatants were subjected to ultracentrifugation (100,000 g, 1 hr, 4°C) to yield membrane and cytosol fractions. Membrane pellets were dissolved in cold NP-40 lysis buffer. Protein concentrations in fractions were determined by BCA assay (Pierce) to normalize samples prepared for SDS-PAGE.

## Authors' contributions

MH performed the simulations and drafted the manuscript. SY carried out the immunoassays. MARS performed the electron microscopy. JE and BW conceived of the study and participated in its design and coordination. All authors read and approved the final manuscript.
